# Development of antibodies against recombinant staphylococcal enterotoxin B from food poisoning cases

**DOI:** 10.14202/vetworld.2024.131-135

**Published:** 2024-01-18

**Authors:** Hidayatun Nisa Purwanasari, Siti Isrina Oktavia Salasia, Fatkhanuddin Aziz, Madarina Wasissa, Fajar Budi Lestari, Christin Marganingsih Santosa

**Affiliations:** 1 Department of Clinical Pathology, Faculty of Veterinary Medicine, Universitas Gadjah Mada, Yogyakarta, Indonesia; 2 Department of Bioresources Technology and Veterinary, Vocational College, Universitas Gadjah Mada, Yogyakarta, Indonesia

**Keywords:** antibody, enzyme-linked immunosorbent assay, recombinant, staphylococcal enterotoxin B, *Staphylococcus aureus*

## Abstract

**Background and Aim:**

Staphylococcal enterotoxin B (SEB) is the most common serotype involved in food poisoning. The aim of this study was to develop immunoassay detection methods using a recombinant enterotoxin B antigen protein to produce recombinant polyclonal antibodies *in vivo*.

**Materials and Methods:**

*Staphylococcus aureus* isolated from a food poisoning case (strain JH5800) was analyzed by polymerase chain reaction (PCR) and confirmed to contain a *seb* gene of 477 bp. A SEB segment was amplified, cloned, sequenced, and aligned. The PCR product corresponding to the predicted mature SEB peptide was inserted into *Escherichia coli* BL21 (DE-3) expression vector and expressed as a hexahistidine-SEB fusion protein. Antiserum against recombinant SEB protein was produced by immunization of Balb/c mice.

**Results:**

In the indirect enzyme-linked immunosorbent assay (ELISA), the polyclonal antibodies produced had a titer of 1:3200. The *seb* gene of *Staphylococcus aureus* isolated from a poisoning case (JH5800) had a molecular size of about 477 bp and a band of recombinant SEB toxin was observed at approximately 30 kDa on SDS-PAGE gel. The polyclonal anti-SEB antibody titer, as revealed by indirect ELISA, was 1:3200 at 59 days.

**Conclusion:**

SEB recombinant protein could be used to produce polyclonal antibodies. ELISA and Western blotting were used to analyze the specificity and sensitivity of the recombinant polyclonal antibodies. Polyclonal antibodies produced could be used to detect SEB on a large-scale.

## Introduction

*Staphylococcus aureus* is a major bacterial pathogen that causes food poisoning outbreaks and various diseases in humans and animals worldwide [1]. *S. aureus* has been designated as the main bacterial causative agent of both clinical and subclinical mastitis in dairy herds [2, 3]. The virulence potential of *S. aureus* predominantly relies on the production of protein toxins, including staphylococcal enterotoxins (SEs), which have remarkable tolerance under a wide range of pH, heating, denaturing agents, and proteolytic digestion [4, 5]. It is undegradable in the digestive tract, can pass through the stomach, and attack intestinal cells [6]. The production of enterotoxins causes various diseases, such as pneumonia, toxic shock syndrome, autoimmune diseases, and food poisoning.

Twenty-four SEs have been described based on their antigenicity from Staphylococcal enterotoxin A (SEA) to Staphylococcal enterotoxin like Y (SElY) [7, 8]. Staphylococcal enterotoxin B (SEB) is the most important and responsible serotype involved in food poisoning among these enterotoxins. Low quantities and concentrations of SEB in food may cause illness. In the immunological detection of the five classical SEs, the predominance of SEB has been observed to be most frequently associated with staphylococcus food poisoning outbreaks in more than 80% of the country [9, 10]. The presence of SEB is commonly associated with the presence of multiple enterotoxins.

To assess the safety of food commodities and confirm the diagnosis in cases of food poisoning, a rapid, accurate, and sensitive test method for detecting the presence of SEs in milk and food at low levels is urgently needed. There is a need to develop a rapid detection tool for the detection of enterotoxins at low concentration. Enterotoxins in the form of mature proteins are antigenic; therefore, serological tests can be developed and used for the analysis of food suspected to contain this toxin.

This study aimed to develop *in vivo* immunoassay detection methods using the recombinant antigen protein of enterotoxin B to produce recombinant polyclonal antibodies. In this study, protein recombinant SEB was used as an antigen to produce polyclonal antibodies by immunization of Balb/c mice. Antibodies against recombinant SEB were produced to develop a sensitive and reliable diagnostic method for SEB detection using indirect enzyme-linked immunosorbent assay (ELISA).

## Materials and Methods

### Ethical approval

In the present study, Balb/c mice were used to produce polyclonal antibodies against SEB. We used three groups of Balb/c mice (25–30 g; 7–8 weeks old) obtained from the Unit for Animal Experiment Services, University of Gadjah Mada, Indonesia. All procedures performed on animals in this study complied with the ethical clearance issued by the Animal Ethics Committee of Universitas Gadjah Mada with registration number 112/EC-FKH/Int./2023.

### Study period and location

This study was conducted from January to October 2023 in Laboratory of Clinical Pathology Universitas Gadjah Mada.

### DNA extraction and amplification of the *seb* gene

SEB-producing *S. aureus* strain JH5800 (concentration 34.1 ng/l) was used to amplify the toxin gene for cloning and expression. Genomic DNA was extracted and purified from *S. aureus* isolated from a dairy cow using DNeasy mini kit (Qiagen, Hilden, Germany) according to the manufacturer’s protocol. Bacterial strains were cultivated on a blood agar base (Oxoid, Germany) for 24 h at 37°C. A total of 5–10 *S. aureus* colonies were suspended in 180 μL TE buffer (10 mM Tris-HCl and 1 mM EDTA, pH 8.0) containing 5 L lysostaphin (1.8 U/μL; Sigma, USA) in 2 mL microfuge tubes. The suspension was incubated for 1 h at 37°C, and 25 μL of proteinase K (14.8 mg/mL; Sigma-Aldrich, Carlsbad, CA, USA) and 200 μL of AL buffer (containing reagents AL1 and AL2; Qiagen) were added. Subsequently, the suspensions were incubated for 30 min at 56°C and then for 10 min at 95°C before being spun at 6,000× *g* for a few seconds. A total of 420 μL of ethanol was added to each sample and placed in a spin QIAmp column. The suspensions were centrifuged at 6,000× *g* for 1 min. The spin columns were placed in a clean collection tube, and the sample was washed twice with 500 μL of AW buffer (Qiagen). After the second wash and centrifugation at 6,000× *g* for 3 min, the QIAamp spin columns were placed in a clean 2 mL microfuge tube, and the DNA was eluted twice with 200 μL and 100 μL of AE buffer (Qiagen). The DNA was stored at −20°C.

The presence of *seb* from the isolate was confirmed using forward sequence primer (5′-TC GCATCAAACTGACAAACG-3′) and reverse primer (5′GCAGGTACTCTATAAGTGCCTGC-3′) [11]. The polymerase chain reaction (PCR) conditions for amplification were optimized as follows: initial denaturation at 95°C for 15 min followed by 35 cycles of denaturation (95°C for 30 s), annealing (57°C for 90 s), extension (72°C for 90 s), and final extension at 72°C for 10 min [11]. The amplified fragment of the *seb* gene was separated using 1.5% agarose gel electrophoresis (Invitrogen, Carlsbad, CA, USA) and stained with RedSafe^®^ nucleic acid staining solution (iNtRon Biotechnology, Korea). The PCR program was run using a Mastercycler^®^ PCR thermocycler (Eppendorf, USA). The amplified PCR product was detected by electrophoresis in 1× TBE buffer at 100 V for 10 min. The resulting bands were visualized using a transilluminator.

### Cloning and expression of *seb* gene into *Escherichia coli*

KAPA HiFi HotStart ReadyMix (KAPA system, USA) was used to amplify the PCR product corresponding to the predicted mature form of SEB peptide with the forward sequence primer (5′-GGAATTCCATATGCACCACCACCACCACCA CGAGAGTCAACCAGATCCTAAAC and reverse (5′-CGCCACGGATCCTCACTTTTTCTTT GTCGTAAG-3′) [11].

The PCR products were digested with *Nde*I and *Bam*HI before ligating the restriction site of pET 22b vector expression (Novagen, EMD Millipore, USA). We transformed the plasmid into the *E. coli* bacterial strain DH5α using standard procedure. Transformed cells were plated on Luria-Bertani broth (10 g NaCl, 10 g Trypticase peptone, and 5 g yeast extract per liter [pH 7.2]) containing ampicillin (100 μg/mL) and incubated overnight at 37°C. The culture plates were stored at 4°C. Insertion was verified by DNA sequencing (ABI 3100 × l; Applied Biosystems, CA, USA).

### Expression of *seb* gene

Recombinant proteins were expressed in *E. coli* BL21 (DE-3) containing the SEB plasmid construct. Bacteria were grown to an optical density of 0.5–0.6 at 600 nm by aeration at 37°C in a 300 mL LB medium containing 100 μg/mL ampicillin as previously described. Cultures were incubated with 0.5 mM (final concentration) isopropyl-β-D-thiogalactopyranoside (IPTG; Sigma-Aldrich Corporation, Carlsbad, CA, USA) and incubated with constant rotation at 30°C for 24 h. The cells were pelleted by centrifugation (4,000× *g* at 4°C for 15 min) and maintained at −80°C until required. Cell pellets were thawed in lysis buffer (5 mM imidazole, 50 mM NaH2PO4, and 300 mM NaCl [pH 7.0]) and lysed by sonication on slurry ice to prevent recombinant protein degradation. The cell pellet was removed by centrifugation (9,000× *g* at 4°C for 20 min), and the supernatant was collected using CO2+ affinity chromatography (Clontech Laboratories, Inc., Mountain View, USA) according to the manufacturer’s protocols for isolation of N-terminally 6His-tagged SEB fusion proteins. Protein purity was determined by 12% sodium dodecyl sulfate-polyacrylamide gel electrophoresis (SDS-PAGE), and proteins were stained with Coomassie brilliant blue R250 (Nacalai Tesque, Kyoto, Japan). Protein concentrations were measured with bovine serum albumin (BSA; Sigma-Aldrich, St. Louis, MO, USA) using a Bio-Rad protein assay (Bio-Rad, Hercules, CA, USA). Purified proteins were dialyzed against Dulbecco’s phosphate-buffered saline (2.68 mM KCl, 1.46 mM KH2PO4, 136.9 mM NaCl, 8 mM Na2HPO412H2O, pH 7.4) at 4°C for 24 h.

### Production of polyclonal antibody

Antibodies were produced by immunization of female Balb/c mice (8 weeks old, 30 g body weight). Adult female mice were repeatedly immunized with 10 g of the recombinant SEB protein. Antigens were emulsified at weekly intervals with an equal volume of Freund’s complete adjuvant for the first injection and Freund’s incomplete adjuvant for the three subsequent injections. Blood samples were collected from the retro-orbitalis plexus 1 day before the primary injections and 1 week after the last injections. Blood samples were incubated at 37°C for 1 h and centrifuged at 4000× *g* at 27°C. The antiserum samples were collected and stored at −20°C until required. After immunization, we measured the immune responses of the mice by indirect ELISA.

### Determination of the antibody against recombinant SEB

The specificity of the produced immunoglobulin G (IgG) against recombinant SEB protein was determined by Western blotting using recombinant IgG as a polyclonal antibody.

## Results

**Figure-1:**
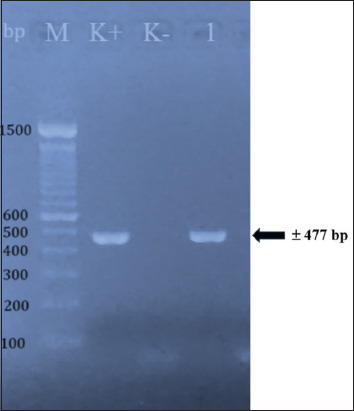
Polymerase chain reaction amplification of *seb* gene of *Staphylococcus aureus* with the size of approximately 477 bp. M = marker, lane K+ = control positive strain, lane K- = control negative strain, lane 1 = *S. aureus* strain US19C.

**Figure-2:**
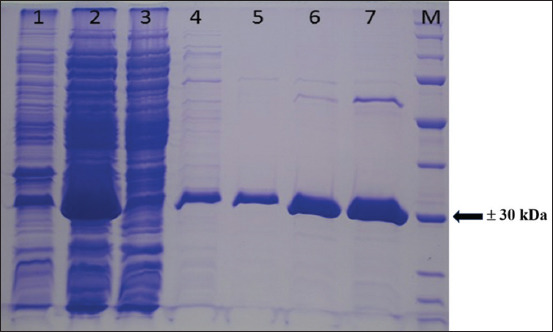
Sodium dodecyl sulfate-polyacrylamide gel electrophoresis of affinity purified recombinant Staphylococcal enterotoxin B. Lane 1= pellet, lane 2 = supernatant, lane 3 = column flowthrough, lane 4–6 = equilibration with 10 mL imidazole 5 mM, 10 mM, 20 mM, respectively, lane 7 = eluate imidazole 200 mM, M = molecular weight marker.

**Figure-3:**
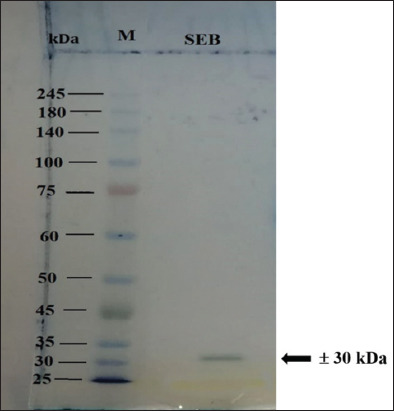
Purified recombinant SEB on Sodium dodecyl sulfate-polyacrylamide gel electrophoresis. M = molecular weight marker, lane 1 purified recombinant SEB with a molecular weight of ± 30 kDa. SEB=Staphylococcal enterotoxin B.

In this study, the *seb* gene of *S. aureus* isolated from a food poisoning case (JH5800) was detected by PCR with a size of approximately 477 bp (Figure-1). Next, we amplified *seb* gene using a designed primer set and the product was ligated into the pET 22b+ vector. PCR-positive clones were analyzed for expression, and a band of recombinant SEB toxin was observed at approximately 30 kDa on SDS-PAGE gel (Figures-2 and 3).

The purified protein recombinant SEB was also used to raise polyclonal antibodies in Balb/c mice models. Balb/c mice were immunized with SEB in a series of four doses. The serum was titrated before injection. The antibody titer is the final serum dilution at which the antibody is detectable. It is defined as the lowest serum concentration at which an antibody assay still produces a detectable positive result. The higher the concentration of antibodies in the blood, the more significant the dilution that will produce a detectable signal. Indirect ELISA observed antibody titers of the obtained serum with antigen (SEB)-coated microtiter plates. Different dilutions of antiserums were added to the SEB-coated wells and secondary conjugates with peroxidase were added. The polyclonal anti-SEB antibody titer, as revealed by indirect ELISA, was 1:3200 on day 59.

**Figure-4:**
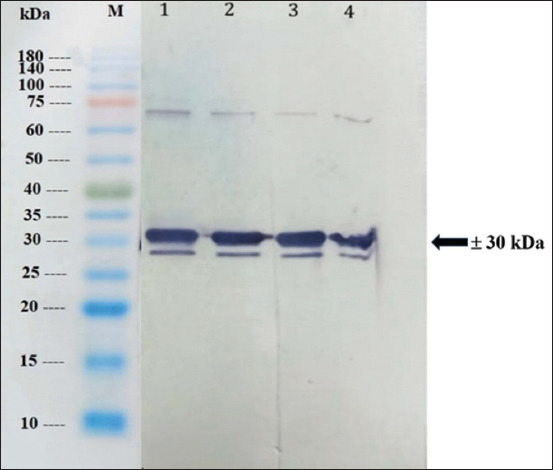
Specificity analysis of polyclonal antibody against SEB by Western blotting M = molecular weight marker, lane 1–4 = antibody against SEB recombinant. The arrow indicates specific protein of staphylococcal enterotoxin B with a molecular weight of approximately 30 kDa. SEB=Staphylococcal enterotoxin B.

Anti-SEB mouse antiserum was subjected to immunoblot analysis to evaluate antibody specificity. Antigen recombinant SEB (3.11 μg) was applied to 12% SDS-PAGE at 10× dilution. Proteins were incubated with a polyclonal antibody (dilution 1:3500) against SEB. After thorough washing, an anti-mouse IgG alkaline phosphatase-conjugated secondary antibody (1:3000, Sigma) was added. Reactions were visualized using a 1-StepTM NBT/BCIP (Thermo Scientific, Waltham, MA, USA). A specific band of approximately 30 kDa, which is characteristic of Staphylococcus enterotoxin B [12, 13], was detected in the antiserum (Figure-4).

A polyclonal antibody against recombinant SEB was produced. An indirect ELISA test was used to determine the optimal concentration of IgG for the detection of SEB. The indirect ELISA results showed a titer of 1:3200. Western blotting analysis showed that the polyclonal antibody specifically reacted with recombinant SEB with a strong band of approximately 30 kDa.

## Discussion

SEs rank second among the causal agents of food poisoning worldwide [12]. The transmission of SEs is unavoidable since bacterial infection may occur in animal farms, slaughterhouses, and veterinary hospitals. In addition, the interconnectedness of animals, in particular livestock and dairy products, the environment, and humans, may increase the possibility of massive transmission. Highlighting the ability to detect low quantities, this research is promising as a new prevention strategy based on one health mission.

As low quantities (100–200 ng) and concentrations (0.5–1 ng/g), SEs can lead to intoxication in food and cause classic food poisoning symptoms (nausea, vomiting, and diarrhea) [13]. SEA, SEB, Staphylococcal enterotoxin C (SEC), and Staphylococcal enterotoxin (SED) are the most common SEs implicated in food poisoning [14, 15]. Toxin concentrations are generally present in various foods, and approximately 0.05–20 ng/g of food and dairy products are frequently involved, with enterotoxins as low as 0.5 ng/g [9, 16]. Therefore, rapid and sensitive detection methods for SEs in food and dairy products are required to confirm the intoxication caused by staphylococcal food poisonings. There is a need to develop more effective tools and procedures to detect and diagnose SPF at this low concentration [9].

ELISA is an immunological method in which an enzyme is used to detect antibodies or antigens in a sample, as well as to examine humoral immune responses. ELISA method can be widely used to detect toxic compounds in food [11]. The indirect ELISA method was developed as a biosensor based on the colorimetric principle as part of the enzyme immunoassay. This method is very sensitive and can be used to detect low concentrations of antibodies [17]. The ELISA is a fundamental tool for detecting toxins such as SEB [18]. Based on the principles of antibody interactions, ELISA allows for easy visualization of results and can be completed without the use of additional radioactive materials. ELISA has become an increasingly important technique in detecting food poisoning agents [11, 19].

There is still a need to develop strategies for detecting SEs. The immunological tests with the production of SEB recombinant protein, which is used to produce polyclonal antibodies [20–23] are required for detection. The development of polyclonal antibodies as biosensors for SEB detection is expected to provide a faster and more sensitive means of detection [24].

## Conclusion

The recombinant SEB can be used to produce polyclonal antibodies. Western blotting was used to analyze the specificity and sensitivity of polyclonal antibodies. The produced polyclonal antibodies can be used to detect SEB on a large-scale.

## Authors’ Contributions

HNP: Performed the experiment, data analysis, and wrote the manuscript. SIOS: Conceptualization, funding acquisition, and supervised and wrote the manuscript. FA: Performed the experiment, data analysis, and reviewed the manuscript. MW: Data analysis and wrote the manuscript. FBL and CMS: Data analysis and reviewed the manuscript. All authors have read, reviewed, and approved the final manuscript.
